# Prevalence and molecular characterization of Glucose-6-Phosphate dehydrogenase deficient variants among the Kurdish population of Northern Iraq

**DOI:** 10.1186/1471-2326-10-6

**Published:** 2010-07-05

**Authors:** Nasir Al-Allawi, Adil A Eissa, Jaladet MS Jubrael, Shakir AR Jamal, Hanan Hamamy

**Affiliations:** 1 Department of Pathology, College of Medicine, University of Dohuk, Azadi Hospital road, 1014 AM Dohuk, Iraq; 2 Scientific Research Center, University of Dohuk, Dohuk, Iraq; 3 Department of Genetic Medicine and Development, Geneva University Hospital, Geneva, Switzerland

## Abstract

**Background:**

Glucose-6-Phosphate dehydrogenase (G6PD) is a key enzyme of the pentose monophosphate pathway, and its deficiency is the most common inherited enzymopathy worldwide. G6PD deficiency is common among Iraqis, including those of the Kurdish ethnic group, however no study of significance has ever addressed the molecular basis of this disorder in this population. The aim of this study is to determine the prevalence of this enzymopathy and its molecular basis among Iraqi Kurds.

**Methods:**

A total of 580 healthy male Kurdish Iraqis randomly selected from a main regional premarital screening center in Northern Iraq were screened for G6PD deficiency using methemoglobin reduction test. The results were confirmed by quantitative enzyme assay for the cases that showed G6PD deficiency. DNA analysis was performed on 115 G6PD deficient subjects, 50 from the premarital screening group and 65 unrelated Kurdish male patients with documented acute hemolytic episodes due to G6PD deficiency. Analysis was performed using polymerase chain reaction/restriction fragment length polymorphism for five deficient molecular variants, namely G6PD Mediterranean (563 C→T), G6PD Chatham (1003 G→A), G6PD A- (202 G→A), G6PD Aures (143 T→C) and G6PD Cosenza (1376 G→C), as well as the silent 1311 (C→T) mutation.

**Results:**

Among 580 random Iraqi male Kurds, 63 (10.9%) had documented G6PD deficiency. Molecular studies performed on a total of 115 G6PD deficient males revealed that 101 (87.8%) had the G6PD Mediterranean variant and 10 (8.7%) had the G6PD Chatham variant. No cases of G6PD A-, G6PD Aures or G6PD Cosenza were identified, leaving 4 cases (3.5%) uncharacterized. Further molecular screening revealed that the silent mutation 1311 was present in 93/95 of the Mediterranean and 1/10 of the Chatham cases.

**Conclusions:**

The current study revealed a high prevalence of G6PD deficiency among Iraqi Kurdish population of Northern Iraq with most cases being due to the G6PD Mediterranean and Chatham variants. These results are similar to those reported from neighboring Iran and Turkey and to lesser extent other Mediterranean countries.

## Background

Glucose-6-Phosphate Dehydrogenase (G6PD) is a key enzyme in the pentose monophosphate pathway and provides the NADPH essential for a number of biosynthetic and detoxifying reactions. The gene encoding G6PD is located at Xq28, and its deficiency is the most common enzymopathy in humans. As an X-linked recessive trait, it is predominantly a disease of males affecting an estimated 400 million people worldwide [1]. This deficiency is associated with a variable spectrum of clinical manifestations including the following: favism, neonatal jaundice, drug induced hemolysis, chronic non-spherocytic hemolytic anemia and infection induced hemolysis [2]. The latter variability is suggestive of biochemical heterogeneity. More than 400 G6PD biochemical variants have already been described [1]. More recently cloning and sequencing of the G6PD gene allowed researchers to characterize more than 140 molecularly distinct variants [3].

G6PD deficiency is prevalent in many countries in the Eastern Mediterranean Region including Iraq [4-8]. Some studies have addressed the prevalence of G6PD deficiency in central and southern Iraq [8-10], but none are available from the northern region. The population of northern Iraq consists of a majority of ethnic Kurds unlike other parts of the country where Arabs predominate. Kurds constitute the fourth largest ethnic group in the Eastern Mediterranean after Arabs, Persians and Turks. They inhabit a region called Kurdistan covering adjacent parts of Iraq, Iran, Turkey and Syria. The origin of Kurds has been a subject of speculation. Based on cultural and linguistic evidence, some historians believe that Kurds are predominantly an Indo-European ethnic group who had migrated and settled in Iran, Iraq and Turkey more than 2000 years ago [11]. However the region has been inhabited by earlier populations since prehistoric times and has been ruled throughout its long history by several major powers including the Assyrians, Persians, Greeks, Romans, Arabs and Turks [12]. All these factors may have contributed to the genetic makeup of the present-day Kurds.

Genetic studies including those of inherited blood disorders are very scarce on Kurds, and such studies may help explain the origin and spread of these disorders and may also help shed some light on the history of this ethnic group. No study of significance has addressed the frequency of G6PD deficiency in the Kurdish Iraqi population, and none has defined the deficient molecular variants among any group of Iraqis, despite the fact that favism is a commonly encountered health problem throughout the country.

This study aims to: 1) determine the prevalence of G6PD deficiency in a random sample of healthy Iraqi Kurdish males attending a regional premarital screening center, and 2) define the molecular basis of G6PD deficiency among a sample of G6PD deficient males in the same region.

## Methods

The study was conducted in the period between July and December 2009. The subjects enrolled were males attending the Central laboratory in Dohuk, northern Iraq for routine premarital health screening. This laboratory serves a population of around one million of mainly ethnic Kurds, and is the only facility in this province that is authorized to provide government mandated premarital investigations. The latter currently include investigation for blood groups, HBsAg, HIV and carrier screening for specific hemoglobinopathies. The center receives on average around 30-40 couples per day. The study subjects were the male partners of the first 10 couples attending the center on every other working day throughout the study period.

A 6 ml blood sample was collected in K2-EDTA tubes from each enrollee. The sample was used to screen for G6PD deficiency by methemoglobin reduction test [13]. All those identified as G6PD deficient and an equal number of age matched non-deficient individuals were further tested by quantitative enzyme assay according to the manufacturer instructions (Biolabo - France). Confirmed deficient individuals were recalled for molecular testing.

Additionally, 65 unrelated male patients who had documented hemolytic episodes associated with G6PD deficiency, were also recalled and had their G6PD deficient status reaffirmed by quantitative enzyme assay.

All G6PD deficient individuals had their DNA extracted by a phenol-chloroform based method [14]. The extracted DNA was screened sequentially for five G6PD deficient mutations namely G6PD Mediterranean (563 C→T), G6PD Chatham (1003 G→A), G6PD Cosenza (1376 G→C), G6PD Aures (143 T→C), G6PD A- (202 G→A) and for silent (1311 C→T) mutation using polymerase chain reaction/restriction fragment length polymorphism (PCR/RFLP) based method. These five deficient variants were selected for screening since they were the five most common variants reported in Iraq's surrounding countries [5,6,15-19]. The primers, amplified exons and restriction enzymes used for each of these reactions are outlined in table 1. Further procedure details were performed as previously reported [20-23].

**Table 1:** The primer sequences, exons amplified and the restriction enzymes used in the PCR/RFLP procedures to detect five G6PD deficient mutations and the silent one [20-23]

Variant	Primers	Exon amplified	Restriction Enzyme
Mediterranean (563 C→T)	**5'GGT GAG GCT CCT GAG TAC CA 3'**	*VI*	*Mbo*II
	**5'AGC TGT GAT CCT CAC TCC CC 3'**		

Chatham (1003 G→A)	**5'CAA GGA GCC CAT TCT CTC CCT T 3'**	*IX*	*Bst*XI
	**5'TTC TCC ACA TAG AGG AGG ACG GCT GCC AAA GT3'**		

A- (202 G→A)	**5'CGT GTC CCC AGC CAC TTC TA 3'**	*III-V*	*Nla*III
	**5' CAC GCT CAT AGA GTG GTG GG 3'**		

Cosenza (1376 G→C)	**5'GCA GCC AGT GGG ATC AGC AAG 3'** **5' GGC AAG GAG GGT GGC CGT GG 3'**	*XI-XIII*	*Bsu*361

Aures (143 T→C)	**5' CAG CCA CTT CTA ACC ACA CAC Ct 3'** **5' CCG AAG TTG GCC ATG CTG GG 3'**	*III*	*Mbo *I

Silent (1311 C→T)	**5' TGT TCT TTC AAC CCC GAG GAG T 3'** **5' AAG ACG TCC AGG ATG AGG TGA TC 3'**	*Part X-XI*	*Bcl *I

Informed consent was taken form all enrollees or their guardians and the study was approved by the appropriate ethical committee at the Dohuk college of Medicine, Iraq.

Statistical analysis utilized the Mann Whitney U test and Chi Squared test wherever appropriate. A p < 0.05 was considered significant.

## Results

Among 580 Kurdish male premarital screening subjects, 63 individuals (10.9%) were found to be G6PD deficient by methemoglobin reduction test with all subsequently being confirmed as deficient by quantitative enzyme assay. Table 2 shows the G6PD enzyme levels in the deficient individuals as well as an equal number of age matched G6PD non-deficient males.

**Table 2:** Mean and standard deviation (SD) of G6PD enzyme concentrations (IU/g Hb) in the two G6PD deficient groups and healthy non-deficient males

Category	No	G6PD Enzyme concentration (IU/g Hb)	
		
		Mean	SD
Group A: G6PD deficient healthy individuals detected by premarital screening	63	0.6670	0.445

Group B: G6PD deficient patients with history of hemolytic episode(s).	65	0.6385	0.443

G6PD non-deficient healthy individuals	63	12.206	0.827

No significant difference were observed in the enzyme concentration between the two deficient groups (p = 0.744)

Among the 63 G6PD deficient males detected, 50 responded to a recall for molecular characterization. Additionally, 65 previously diagnosed males with G6PD deficiency associated documented hemolytic episodes also underwent quantitative enzyme assays (table 2) and molecular investigation. Molecular characterization of these 115 G6PD deficient individuals revealed that the G6PD Mediterranean (563 C→T) was the most common and was detected in 101 of 115 individuals (87.8%), followed by G6PD Chatham (1003 G→A) that was detected in 10 (8.7%). No cases with G6PD A-, Aures or Cosenza were identified. This left four cases (3.5%) uncharacterized. There were no significant differences in the distribution of molecular G6PD variants among G6PD deficient individuals detected by screening and those with previously documented hemolytic episodes (table 3). On the other hand, the mean enzyme concentration of subjects with Mediterranean variant was significantly lower at 0.5798 + 0.357 IU/g Hb than subjects with the Chatham variant at 0.846 + 0.3299 IU/g Hb, P = 0.029.

**Table 3:** The distribution of the G6PD deficient molecular variants in asymptomatic individuals detected by premarital screening and in patients with history of hemolytic episodes in the current study

	Number of cases (%)		
**Category (no.)**	**Mediterranean**	**Chatham**	**Uncharacterized**

G6PD Deficient healthy individuals (50)	45 (90)	3 (6)	2 (4)

G6PD Deficient patients (65)	56 (86.2)	7 (10.8)	2 (3.1)

Total (115)	101(87.8)	10 (8.7)	4 (3.5)

The silent mutation (1311 C→T) was documented in 93/95 (97.8%) of the Mediterranean deficient variants, in 1/10 of the Chatham cases and in one of the three uncharacterized variants.

## Discussion

G6PD deficiency has long been recognized as a common inherited hematological disorder in Iraqis including Kurds [7,8]. Kurds make up about 20% of the population of Iraq, and they live in Northern and Northeastern Iraq. The area covered by the current study in northern Iraq lies at the center of the Kurdish inhabited areas of the Eastern Mediterranean Region.

Earlier limited studies on G6PD deficiency among Iraqi Kurdish males living in Baghdad (at the center of the country) have reported frequencies of 7.6-8.8% [7,9], which is slightly lower than the frequency of 10.9% as documented in this study. The latter rate is also higher than rates reported from the neighboring Kurdish population of western Iran [15], but is remarkably less than reports from Kurdish Jews, who have one of the highest rates worldwide of up to 58% [24]. Previous studies from other parts of Iraq revealed variable results from Baghdad (6.1-12.4%) at the center of country and 15.3% from Basra at the extreme south [7-10]. As outlined in Table 4, polymorphic frequencies of G6PD deficiency were reported throughout the Eastern Mediterranean Region ranging from 2% in Lebanon to 39.8% in Eastern Saudi Arabia [4-10,15,25,26]. The polymorphic frequency of G6PD deficiency in Iraq and in the Eastern Mediterranean Region may be linked to selective advantage against malaria in these previously highly endemic areas [1,27]. Northern Iraq was highly endemic in Malaria up to the fifties and sixties of the last century when control programs sponsored by the WHO managed to control the disease, but it remerged in the nineties particularly in the area covered by this study [28-30].

**Table 4:** Prevalence rates of G6PD deficiency in Iraq and some of the surrounding countries in Eastern Mediterranean Region

Country	Prevalence (%)	Ref
Iraq: Central (Baghdad)	6.1-12.3	[7-9]

Iraq: Southern (Basra)	15.3	[10]

Iraq: Northern (Kurds)	10.9	Current

Iran: Western (Kurds)	5.3	[15]

Jordan	3.6	[4]

Kuwait	5.5	[4]

Saudi Arabia	1-39.8	[25,45,46]

Syria	3.0	[4]

Turkey	0.5-20	[26]

Bahrain	18	[47]

Egypt	5.9	[48]

Lebanon	2.1	[4]

Oman	2-29	[49]

United Arab Emirates	11	[50]

The predominance of the Mediterranean mutation (563 C→T) as demonstrated in the current study, has been documented by many studies from the Eastern Mediterranean countries, where it constitutes 54 to 91% of G6PD variants identified (table 5) [5,6,15-19]. Our figures are comparable to those reported in Kurds from neighboring western Iran or from Kurdish Jews of 80-97% [15,24]. It is important to note that based on biochemical characterization, the Mediterranean variant has been presumably detected in 58.6% of 31 G6PD deficient males from an earlier study in Baghdad [9]. However, the small number of biochemically characterized deficient variants in the latter study and the fact that biochemical phenotypes are not absolutely related to particular molecular variants [31] would limit the feasibility of making reliable comparisons with the current study. The ethnic background of Baghdad's population is different from that of northern Iraq, thus the presumed lower frequency of the Mediterranean mutation maybe actual but would require molecular studies to be verified. The G6PD Mediterranean variant is also the predominant mutation among other Mediterranean countries like Italy and Greece as well as in the Indian subcontinent, but it is virtually non-existent in Central and South Africa and it shows a decreasing frequency as we move to the east from India (table 5) [32-38]. As with other Mediterranean and Middle Eastern countries, the large majority of those with G6PD Mediterranean carry the silent 1311 C→T mutation, and only 2 of the 95 cases tested in this study were non-carriers, the latter maybe due an intragenic recombinant event or population admixture [15,39,40]. Tishkoff and coworkers after studying some highly polymorphic microsatellites and RFLPs in and around the G6PD gene proposed that the Mediterranean mutation is of rather recent origin, originating somewhere in the Mediterranean basin within the past 1600 to 6640 years. Thereafter, it was spread to Middle East and North Africa by extensive trade routes and colonization by the Greeks in the first millennium BC. The pattern of distribution of the Mediterranean mutation (with the silent 1311 T mutation) makes it quite conceivable that it may have spread through the army of Alexander the Great who conquered the Middle East and North Africa and went as far east as India [41]. The Kurdistan region was a path which he used to pursue his conquests to Persia and beyond [12]. The mutation, thereafter, may have been selected for by malaria already highly endemic in these fertile agricultural lands, particularly around 500BC and thereafter. It is important to note that the Mediterranean mutation has also been reported predominantly without the silent mutation (i.e. with 1311 C) in the Indian subcontinent and southern Italy, which led many investigators to suggest two independent origins of the Mediterranean mutation [34,39,42].

**Table 5:** The distribution of five G6PD deficient variants (given as percentages) in different populations

Population	No.	Deficient G6PD Variants (%)					Ref
			
		Mediterranean	Chatham	A-	Aures	Cosenza	
Iraq: Northern (Kurds)	115	87.8	8.7	-	-	-	Current

Iran: Western (Kurds)	68	91.2	7.3	-	-	1.5	[15]

Iran: Northern (Mazandran)	74	66.2	27	-	-	6.75	[16]

Iran: Southern (Fars)	74	83.8	13.5	1.35	-	-	[17]

Jordan	28	53.6	3.6	14.2	3.6	-	[18]

Kuwait	89	74.2	10.1	12.4	3.0	-	[19]

Saudi Arabia: Eastern Province	114	84	-	5.8	-	-	[6]

Turkey	50	80	4	2	-	-	[5]

Italy	162	69	-	3.7	-	1.2	[32]

India	167	60.4	1.2	-	-	-	[34]

Malaysia: Malays	86	26.7	2.3	-		-	[35]

Algeria	100	23	1	46	7	1	[22]

Nigeria	89	-	-	100	-	-	[36]

The second most frequent variant detected by the current study is G6PD Chatham (1003, G→A) seen in 8.7% of deficient individuals. G6PD Chatham, although first described in a patient of Asian Indian origin, is now recognized as one of the common variants worldwide [15]. Table 5 outlines the frequencies of the latter mutation in various Eastern Mediterranean countries and shows rather comparable figures among Iranian Kurds in western Iran [15]. However, figures for its frequency vary in other parts of the latter country reaching 27% in Northern Iran [16,17]. This variant may have been introduced to northern Iraq through gene flow from neighboring Iran.

G6PD Cosenza (1376 G→C) was first described in Southern Italy [43] and has been reported in variable frequencies by some Iranian studies including the one on Kurds [15,16]. However, this variant was not detected in any deficient individual in the current study. G6PD Aures (143 T→C) was first described in Algeria [22]. Since then it has also been reported in variable frequencies in Jordan, Kuwait and western Saudi Arabia [18,19,44]. However it was not reported among Iranian Kurds or in neighboring Turkey [15,26] as is the case in the current study.

The African A-variant (202 G→A) was not detected among our sample. This is in contrast to an earlier report from central Iraq where it was reported in 4.3% of G6PD deficient subjects [9] and in some Arab Eastern Mediterranean countries where rates between 5.8-14.2% were given (table 5). However, and similar to the current study, absence of the variant has also been described in reports on Iranian Kurds and Northern Iranians [15,16]. Absence of the variant in these areas is most likely related to the fact that the Kurdish inhabited areas of Iraq and neighbouring Iran were, unlike Arabia, a much less likely destination of African gene flow prior to or during the reign of the prosperous Islamic empire.

## Conclusion

This study has documented that the frequency of G6PD deficiency among the predominantly Kurdish population in Northern Iraq is 10.9%, which is higher than that reported from the neighboring Iranian Kurdish population. The study which is the first molecular study on G6PD deficient variants from Iraq documented that G6PD Mediterranean and Chatham constitute the large majority of the deficient variants which is similar to findings reported in surrounding populations. Further studies including larger number of patients, more diverse ethnic backgrounds and including screening for other deficient variants and DNA sequencing are needed to give a more comprehensive view of G6PD variants in Iraqis.

## Competing interests

The authors declare that they have no competing interests.

## Authors' contributions

NA, contributed the concept and design, part of the molecular studies, data analysis and drafting of the manuscript; AAE, contributed to the collection of data, larger part of the molecular work and analysis of data; JMSJ, contributed to the concept and design and part of the molecular work; SARJ, contributed to collection of data, performing hematological, enzyme assays and data analysis; HH, contributed to the analysis and interpretation of results, drafting and revision of the manuscript. All authors revised and approved the final submitted version of the manuscript.

## Pre-publication history

The pre-publication history for this paper can be accessed here:

http://www.biomedcentral.com/1471-2326/10/6/prepub
